# Rationale and design of the Baptist Employee Healthy Heart Study: a randomized trial assessing the efficacy of the addition of an interactive, personalized, web-based, lifestyle intervention tool to an existing health information web platform in a high-risk employee population

**DOI:** 10.1186/s13063-016-1424-z

**Published:** 2016-07-01

**Authors:** Janisse M. Post, Shozab S. Ali, Lara L. Roberson, Ehimen C. Aneni, Sameer Shaharyar, Adnan Younus, Omar Jamal, Rameez Ahmad, Muhammad A. Aziz, Rehan Malik, Erica S. Spatz, Theodore Feldman, Jonathan Fialkow, Emir Veledar, Ricardo C. Cury, Arthur S. Agatston, Khurram Nasir

**Affiliations:** Center for Healthcare Advancement and Outcomes, Baptist Health Medical Group, 1691 Michigan Avenue Suite 500, Miami Beach, FL 33139 USA; University of Manchester School of Medicine, Manchester, UK; Aventura Hospital & Medical Center, Aventura, FL USA; Center for Outcomes Research and Evaluation, Yale School of Medicine, New Haven, CT USA; Miami Cardiac & Vascular Institute, Baptist Health South Florida, Miami, FL USA; Ciccarone Center for the Prevention of Heart Disease, Johns Hopkins University, Baltimore, MD USA; Herbert Wertheim College of Medicine, Florida International University, Miami, FL USA; Robert Stempel College of Public Health, Florida International University, Miami, FL USA

**Keywords:** Metabolic syndrome, Diabetes, Cardiovascular disease

## Abstract

**Background:**

Metabolic syndrome (MetS) and diabetes confer a high risk for developing subsequent cardiovascular disease (CVD). Persons with MetS constitute 24–34 % of the employee population at Baptist Health South Florida (BHSF), a self-insured healthcare organization. The Baptist Employee Healthy Heart Study (BEHHS) aims to assess the addition of a personalized, interactive, web-based, nutrition-management and lifestyle-management program to the existing health-expertise web platform available to BHSF employees in reducing and/or stabilizing CVD and lifestyle risk factors and markers of subclinical CVD.

**Methods/design:**

Subjects with MetS or Type II Diabetes will be recruited from an employee population at BHSF and randomized to either an intervention or a control arm. The intervention arm will be given access to a web-based personalized diet-modification and weight-modification program. The control arm will be reminded to use the standard informational health website available and accessible to all BHSF employees. Subjects will undergo coronary calcium testing, carotid intima-media thickness scans, peripheral arterial tonometry, and advanced lipid panel testing at visit 1, in addition to lifestyle and medical history questionnaires. All tests will be repeated at visits 2 and 4 with the exception of the coronary calcium test, which will only be performed at baseline and visit 4. Visit 3 will capture vitals, anthropometrics, and responses to the questionnaires only.

**Conclusion:**

Results of this study will provide information on the effectiveness of personalized, web-based, lifestyle-management tools in reducing healthcare costs, promoting healthy choices, and reducing cardiovascular risk in an employee population. It will also provide information about the natural history of carotid atherosclerosis and endothelial dysfunction in asymptomatic but high-risk populations.

**Trial registration:**

ClinicalTrials.gov registry, NCT01912209. Registered on 3 July 2013.

## Background

Metabolic syndrome (MetS) is the coexistence of a number of metabolic risk factors that appear to directly promote the development of atherosclerotic cardiovascular disease (CVD) [1]. The increased risk is thought to be greater than that conferred by any of its individual components [2]. Persons with MetS incur higher healthcare costs, even in the absence of CVD events or hospitalizations [3]. The elevated CVD risk and downstream costs incurred by MetS have generated great interest in management and prevention strategies aimed at reducing the overall CVD burden in this population.

With the incidence and prevalence of chronic diseases such as CVD [4, 5] and diabetes [4, 6, 7] on the rise, more organizations are implementing worksite wellness programs. According to a Kaiser/Health Research and Educational Trust survey [8], 92 % of employers with 200 or more employees in the United States offer wellness programs. The percentage of organizations offering wellness programs in 2009 was 58 %, and the recent survey result represents a significant increase. This dramatic increase is attributed to smaller organizations adopting web-based health-promotion programs [8]. The American Heart Association recommends conducting more workplace cardiovascular wellness research involving high-risk populations [9]. Worksite internet-based cardiovascular prevention and wellness programs that focus on diet and lifestyle modification show promise as part of a comprehensive healthcare package to be offered at the workplace. The web-based approach provides the promise of reach to remote workers and those with nonconventional schedules, and can provide an affordable platform for self-managed programs.

Enabling patients to adopt healthier lifestyles remains a cornerstone of prevention strategies for a variety of conditions including MetS. Lifestyle modification is a necessary supplement to clinical therapy in order to achieve and maintain long-term health [10, 11]. A number of methods are used to achieve lifestyle modification, ranging from intensive supervised activities to an occasional reminder by a physician [11]. Recently, web-based tools have been increasingly employed to supplement lifestyle interventions although their effectiveness remains controversial [11, 12]. The effectiveness of online interventions in subjects with MetS in employee populations is less well-studied [12].

Baptist Health South Florida (BHSF) is a large self-insured organization and has a vested interest in the wellness of its employees. BHSF provides a number of tools to its employees in an attempt to reduce CVD risk, including intensive boot camps, weight loss programs [13], and a web-based suite of tools [14–16]. All employees have access to an online suite of wellness tools as a standard of care.

In this context, the present study aims to assess the impact of the addition of a personalized web-based approach to the standard-of-care online tools in order to determine the effectiveness of these tools in improving the cardiovascular health of its employees.

### Study objectives

The primary purpose of BEHHS is to assess if the addition of a personalized, interactive, web-based, diet and lifestyle-counselling system to an existing web-based wellness engine will reduce/stabilize components of the metabolic syndrome and markers of subclinical CVD in an employee population. Primarily, this addition is hypothesized to result in improvement of subclinical markers of cardiovascular disease as a direct impact of expected weight loss in the intervention arm versus the control arm.

The secondary purpose of BEHHS is to determine if the addition of a personalized web-based engine results in a greater adoption of healthy behaviors (including dietary habits, sleep habits, and physical activity levels) compared to a nonpersonalized wellness engine.

Third, the study aims to assess if the addition of a personalized web-based engine to an existing platform results in stabilization of subclinical atherosclerotic disease progression in high-risk patients.

Fourth, the study aims to document the natural history of atherosclerotic disease (as assessed by coronary artery calcification, carotid intima-media thickness, and peripheral artery tonometry) in a high-risk employee population.

## Methods/design

### Study design

**Fig. 1:**
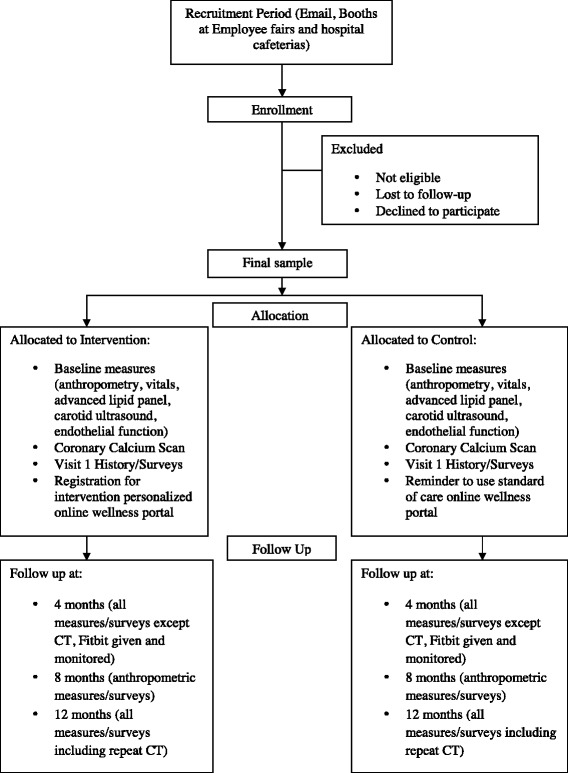
Study flow chart and timeline of events

The Baptist Employee Healthy Heart Study is a prospective, randomized, nonblinded study. The study sample will consist of approximately 200 male and female BHSF employees meeting the inclusion/exclusion criteria for the study. The participants will be randomized into two groups: the intervention arm, Group 1, or the control arm, Group 2 (Fig. 1).

#### Inclusion criteria

Study participants must be current employees of BHSF, have access to the internet, and possess a valid email address. In addition, participants will be required to have a diagnosis of either Type II Diabetes or MetS. MetS is defined as per the updated NCEP-ATP III guidelines [1]. As per guidelines, the diagnosis of MetS requires three or more of the following: waist circumference > 40 inches in men and > 35 inches in women, fasting triglyceride level ≥ 150 mg/dl or Rx, HDL-C < 40 mg/dl in men or < 50 mg/dl in women or Rx, BP > 130/85 mmHg or Rx, and fasting blood glucose ≥ 100 mg/dl or Rx.

#### Exclusion criteria

Exclusion criteria include a previous diagnosis of CVD or history of major cardiovascular events (angina, myocardial, and/or prior coronary revascularization), known history of COPD, atrial fibrillation, hypotension, heart failure, valvular disease, and heart block. BHSF employees currently participating in other BHSF wellness programs for weight loss are considered ineligible. Women of childbearing potential who are pregnant or seeking to become pregnant or currently breastfeeding are excluded, as are women with a history of bilateral mastectomy. Subjects who are receiving active treatment for cancer or had undergone CT scan of the chest during the year prior to their enrollment were also excluded.

Randomization will be performed via computerized methods, and assignment groups will be printed and placed in sealed envelopes by nonstudy staff. On the study visit day, the study staff will open the envelope containing the group assignment and provide study participants with group-specific instructions. The study staff will undergo adequate training to prevent selection bias.

### Description of the intervention program

Subjects randomized to the intervention program will be given an access key to a personalized web-based program. The program includes personalized diet and weight guidance, meal planners, shopping list generator, recipe finder, weight tracker, exercise tracker, dining out, and fast food guides. Participants are able to track their diet with a food journal each day, and enter their weight change over time. The intervention program provides personalized nutrition counseling and weight-loss advice from a registered dietician. The participants receive phone and e-mail access to a dietician. In addition, the interactive web-portal provides weight loss guidance in response to the participant use of the online weight tracker. Participants receive tips and suggestions on how to make changes in their diet and lifestyle each time they log in to the website. Community support is also available to the participants through an online forum comprising other participants of the program. The use of the above-mentioned tools is optional for participants in the intervention program.

### Description of the standard of care online wellness program

Control group subjects will continue to receive access to a web-based infomational health program available to all Baptist Health employees. Participiants will be shown how to access the website and, if needed, will be given information on how to reset their passwords. The program provides educational material regarding healthy diet and physical activity. It also provides health trackers and goal setting for certain health targets.

### Data collection and storage

All anthropometric, clinical examination, and survey data will be collected on standardized case report forms, which are then transferred to an electronic database. The data will be assessed for consistency prior to upload, and a number of quality control and validation checks will be performed within the software to ensure accuracy of the data. EndoPAT data will be obtained via machine-generated excel sheets, which will then be uploaded into the electronic database. An individual coronary artery calcium (CAC) score will be obtained in the form of a radiologist report, which is then entered into the electronic data capture system. Blood testing results will be transferred electronically from the Quest Diagnostics-Nichols Institute to the study center periodically. Fitbit data (physical activity tracker data) will be collected periodically from the Fitbit online portal with the consent of study participants.

### Recruitment

The primary recruitment strategy will consist of sending a series of monthly emails to all BHSF employees informing them of the study. Study information will be placed on the BHSF intranet website, and study flyers and posters will be placed throughout the various BHSF entities. We will also reach potential participants by placing study information in the weekly BHSF employee newsletters. Potential participants will be screened via telephone or email by the study staff using a standardized questionnaire to assess them for study eligibility. Inclusion and exclusion criteria will be assessed based on self-report.

The secondary recruitment strategy will consist of informational tables set up by study staff throughout various BHSF entities, at which, study staff will provide study information to potential participants through presentations. Additionally, staff will provide blood pressure, lipid, and finger-stick glucose screening for potential study participants who are unsure of their lab values.

#### Consent and enrollment

Participants meeting study eligibility criteria will be scheduled for an appointment at the study center. Participants are required to fast for 9–12 hours prior to study procedures. A copy of the consent form will be provided to potential participants prior to the visit. Upon arrival in the clinic, each participant will be asked to review and sign the informed consent.

### Study procedures

The following study procedures are conducted at baseline, month 4, and month 12, with the exception of the coronary artery calcium scoring, which will be repeated only at month 12. The month 8 study visit will capture vitals, anthropometrics, and questionnaire responses only. Table 1 provides a detailed breakdown of procedures that will be conducted at each study visit.

1. Ensuring study eligibility
  At the beginning of the first visit, the study coordinator reviews the participant’s medical history with the participant to ensure study eligibility. After confirming eligibility, participants are then required to review and sign an informed consent form.
2. Measurement of vital signs
  Seated blood pressure: Following 5 minutes of rest in the sitting position, blood pressure is determined in the right arm following the American Heart Association guidelines [17]. Body weight, heart rate, and blood pressure are obtained prior to venipuncture.
  Heart rate: Following 5 minutes of rest in the sitting position, the heart rate is assessed by palpation for 30 seconds and recorded as beats per minutes after multiplying by 2.
3. Urine pregnancy test (if applicable)
  A urine pregnancy test is performed on all female participants of childbearing age to exclude pregnancy. Women who have a positive pregnancy test will be excluded.
4. Anthropometry
  - Body weight: Subjects are weighed, without their shoes, on a calibrated scale after heavy objects (such as keys and coins) have been removed from their pockets. The results are recorded to the nearest 0.2 pounds.
  - Height: Height is measured by standing next to a standardized scale after removing footwear.
  - Abdominal circumference: The subject’s abdomen is cleared of all clothing and accessories. The feet are positioned shoulder width apart and arms crossed over the chest in a relaxed manner. Using the NIH protocol, the waist circumference measurement is taken at the top of the iliac crest using an anthropometric tape. Ensuring that the measuring tape is positioned in a horizontal plane around the abdomen, tension is applied to the tape to ensure a snug fit without causing skin indentation. At the end of a normal expiration, the measurement is taken to the nearest 0.5 cm.
  - Hip measurement: Hip circumference is measured at the level of the greatest girth, which is usually at the level of the trochanters. Measurements are made to the nearest 0.5 cm.
  - Body fat percentage: The bioelectrical impedance method is utilized, with a handheld device being used to calculate body fat percentage (Omron HBF-306C) [18].
5. Venipuncture
  Approximately 30 ml of venous blood will be drawn, labeled, processed, and shipped to Quest Diagnostics for advanced cardiovascular testing, which includes fasting plasma glucose, standard lipid panel testing, and advanced markers. Advanced markers include ApoB, Lp(a), homocysteine, Lp-Pla2, hsCRP, fibrinogen, insulin, NT–proBNP, vitamin D, Omega 3 and 6, and others. A genetic panel that includes KIF6, CYP2C19, ApoE, LPA-Aspirin, LPA-Intron 25, 4q25-AF, and 9p21-MI risk genotypes will also be performed (see Table 2).
6. Carotid intima-media thickness (cIMT) scanning
  The study will employ a Panasonic CardioHealth Station automated carotid ultrasound screening device [19] for fully automated cIMT measurements using a single transducer angle. The mean cIMT measurement is defined as the mean of 24 spatial measurements performed over a 1-cm region in the far wall of the common carotid artery. In addition, atherosclerotic plaque is defined as any obvious focal luminal encroachment > 1.00 mm.
7. Endothelial function testing
  The reactive hyperemia index (RHI) to assess endothelial function and augmentation index (AI) to assess arterial stiffness are measured using the Itamar EndoPAT 2000 system [20]. The test procedure involves a baseline reading with the EndoPAT probes on the index finger for 5 minutes. A blood pressure cuff is then applied to the nondominant arm for the next 5 minutes to occlude blood supply, and a reading is taken during this time. After 5 minutes of occlusion of the nondominant arm, the pressure is released. Blood flow to the arm resumes, which causes blood vessels to dilate, at which time the EndoPAT machine calculates stiffness in the arterial walls. The vessel wall reactivity is a comparison of the pre-occlusion and postocclusion blood flow and is reported as the reactive hyperemia index.
8. Questionnaires
  Participants are asked to complete a series of questionnaires at each study visit (Table 3). The domains assessed will included demographics, past medical history, family history, socioeconomic status, dietary habits, physical activity, current medications (including dose and frequency), and self-perception of health.
9. Coronary artery calcium score (CAC)
  Subjects over the age of 35 undergo CAC scanning. Coronary arteries are scanned by noncontrast multiple detector computed tomography (MDCT) (acquisition time: 100 msec, 3-mm slice thickness) during end-diastole. Coronary calcium is classified with a CT threshold of 130 Hounsfield units (HU) involving three contiguous voxels for identification of a calcific lesion, resulting in a minimum lesion area of 1.02 mm. The average radiation dose is 0.5–1 mSv. The scanning procedures and protocols used in this study are the same as those used in previous studies and follow current Baptist Health South Florida radiology protocols. If a CAC score ≥ 400 is found, the patient and the patient’s primary care physician will be notified of the CT scan results immediately.
10. Fitbit—physical activity tracker
  All participants are provided with a Fitbit pedometer on their second study visit. An online account will be created for them, and login information will be provided to participants to enable them to track their physical activity. Physical activity data is retrieved periodically from the online portal and stored electronically.

**Table 1:** Baptist Employee Healthy Heart Study (BEHHS) procedures

	Visit 1 (Enrollment)	Visit 2 (4 Months)	Visit 3 (8 Months)	Visit 4 (12 Months)
Consent	X			
Vitals	X	X	X	X
Family history/medical history	X			
Urine pregnancy test (if applicable)	X			X
Anthropometry (weight, height, waist and hip circumference, body fat %)	X	X	X	X
Venipuncture	X	X		X
Standard and advanced lipid testing (TC, LDL, HDL, triglycerides, lipoprotein(a), apolipoprotein B, LDL and HDL subclasses)				
EndoPAT	X	X		X
Carotid ultrasound (cIMT)	X	X		X
Demographic questionnaire	X			
Healthy Days and Sleep Questionnaire	X	X	X	X
Healthy Behaviors Questionnaire	X	X	X	X
Smoking and Alcohol Use Questionnaire	X	X	X	X
Medication Use Questionnaire	X	X	X	X
Exercise and Physical Activity Questionnaire	X	X	X	X
CT appointment referral (if applicable)	X			X

**Table 2:** Baptist Employee Healthy Heart Study (BEHHS) genetic testing

Genotype	Clinical utility
KIF6	Provides genetic information about a person’s increased risk for heart disease and reduction of heart disease events with atorvastatin or pravastatin therapy
CYP2C19	Provides genetic information about a person’s metabolism of certain drugs such as clopidogrel
ApoE	Provides genetic information about a person’s increased risk for heart disease and response to different amounts of dietary fat
LPA-Aspirin	Provides genetic information about a person’s increased risk for heart disease and reduction of heart disease events with aspirin therapy
4q25-AF	Provides genetic information about a person’s increased risk for atrial fibrillation and risk for stroke caused by atrial fibrillation
LPA-Intron 25	Provides genetic information about a person’s increased risk for heart disease
9p21-MI	Provides genetic information about a person’s increased risk for a myocardial infarction at an early age, abdominal aortic aneurysm, and coronary heart disease

**Table 3:** Major data points in Baptist Employee Healthy Heart Study (BEHHS) surveys

Survey title	Main data elements
Demographic	Age, ethnicity, family income, education, family size
Healthy Days and Sleep	Physical and mental health status during the past month. Sleep duration and quality.
Diet	Fruit and vegetable consumption, “unhealthy” food consumption (fast food and red meat etc), Mediterranean diet assessment
Smoking and Alcohol Use	Frequency and type of alcohol consumption, smoking history including number of years and quantity smoked
Exercise and Physical Activity	Duration in minutes per week of vigorous and moderate physical activity during work, travel, recreational time. Sedentary time/day
Medical History	History of stroke, TIA, hypercholesterolemia, hypertension, asthma, sleep apnea, others
Family History	Age of diagnosis of diabetes, stroke, myocardial infarction, COPD, PCI/CABG
Medication History	Current medications, dose, frequency and route of administration

### Statistical analysis

#### Sample size calculations

Our hypothesis is that the addition of a personalized, interactive, web-based diet and lifestyle counselling system to an existing, web-based, wellness engine will reduce/stabilize components of the MetS, so differences in values of components of the MetS and markers of subclinical CVD (CVD is not apparent at baseline), will be observed on follow-up visits at 4, 8, and 12 months.

A two-by-two repeated measures design consists of two groups of subjects, each measured at two time points. In this case, the primary goal of the study is to compare the change of weight across time in group 1 to the change across time in group 2. Sample sizes of 85 in group 1 and 85 in group 2 achieve 80 % power to detect a difference in mean weight changes of 5.8 lbs. with a standard deviation of 10.0 lbs. at the first time point, a standard deviation of 10.0 lbs. at the second time point, and a correlation between measurement pairs of 0.100. The significance level (alpha) is 0.05 using a two-sided, two-sample t-test.

Group sample sizes of 85 and 85 achieve 80 % power to detect a difference in BMI of 1.019 kg/m^2^, 1.359 kg/m^2^, or 1.699 kg/m^2^ in a design with four repeated measurements and having a compound symmetry covariance structure when the standard deviation is 3, 4, or 5, the correlation between observations on the same subject is 0.500, and the alpha level is 0.050.

#### Primary analysis

The primary endpoint is BMI reduction and the improvement/stabilization of measures of subclinical CVD. Additionally, we will examine changes in anthropometric indices (waist circumference, waist to hip ratio, and percentage of body fat), metabolic parameters, conventional/advanced lipid testing, inflammatory marker panel, systolic and diastolic blood pressure, dietary and physical activity scores, coronary artery calcification, carotid intima-media thickness, and endothelial function. The primary null hypothesis is that no difference exists between the treatment groups in the outcomes measured.

Analysis will be conducted using the intention-to-treat principle; that is, all subjects who were randomized will be included in the data analysis regardless of completion of the study status. Continuous variables (e.g., age and biochemical variables) will be assessed for normality by examining graphically and by statistical methods. Categorical variables will be described using frequency distributions, whereas continuous variables will be described using means and standard deviations for normally distributed variables, and median (interquartile range) for non-normally distributed variables. Differences relating to study-arm assignment in these variables between groups will be evaluated using chi-square tests for categorical variables and independent t-tests for continuous variables.

### MDCT data analysis

The lesion score will be calculated with the area density method, by multiplying the lesion area by a density factor derived from the maximal Hounsfield unit within the area as described by Agatston and Janowitz [21]. The density factor will be assigned in the following manner: 1 for lesions whose maximal density was 130–199 HU, 2 for lesions 200–299 HU, 3 for lesions 300–399 HU, and 4 for lesions ≥ 400 HU. A total CAC score will be determined by summing the individual lesion scores from each of four anatomic sites (left main, left anterior descending, circumflex, and right coronary arteries).

### Quality control and assurance

All data are subjected to periodic quality control checks by the database administrator and study coordinators. Additionally, all study data is subject to a periodic audit by the Institutional Review Board at Baptist Health South Florida.

### Cohort surveillance and follow-up

Contact information is obtained for each study participant at baseline, and study coordinators contact all study participants to schedule follow-up visits. At each follow-up visit, standardized questionnaires are used to capture pertinent health and lifestyle information.

### Notification of incidental study findings

At each visit, participants will be asked if they or their primary care physicians would like to receive test results. Copies of all study test results (including in-clinic measurements) will be mailed to participants in both arms of the study and their primary care physicians after every study visit. Any incidental findings or conditions that merit urgent medical evaluation (e.g., CAC ≥ 400), will be reported to participants and their physicians via telephone and/or email as soon as they are identified. Additionally, all participants will be able to contact the principal investigator in order to discuss their test results at any time.

## Discussion

The BEHHS is the first randomized trial at BHSF to assess the effectiveness of a personalized, interactive web-based nutrition and lifestyle management program in a high-risk employee population. We hope this will result in the development of a “turn-key” program that could be utilized by employee health groups to improve metabolic health and reduce subclinical cardiovascular disease burden via large-scale, low-cost interventions.

The Baptist Employee Healthy Heart Study (BEHHS) will also allow us to determine potential risk factors involved in the development of subclinical cardiovascular disease in an asymptomatic employee population and, therefore, aid in devising strategies to prevent CVD. The use of internet-based employee wellness programs can present an opportunity to minimize absenteeism and presenteeism and decrease healthcare costs. Baicker et al. indicated that employee wellness programs offer an average of 3.27 USD reductions in medical costs for every dollar spent [22].

The BEHHS study will also permit the determination of interactions between risk factors and the noninvasive assessment of subclinical cardiovascular disease and progression over time to symptomatic disease. It may also give more insight into the pathophysiology of atherosclerosis and provide a more personalized approach to the prevention of atherosclerotic disease. The current models of CVD risk prediction provides limited opportunities to assess the impact of risk-factor modification and lifestyle changes. BEHHS aims to expand the use of CAC and cIMT and their correlation with genetic susceptibility of an individual to atherosclerotic disease, and development of new risk prediction models that would help identify individuals predicted to have a high lifetime risk but are designated as low risk using current models.

The use of advanced noninvasive imaging techniques (CAC and cIMT) in harmony with genetic and advanced lipoprotein testing provides a unique cluster of data to determine prevalence and progression of atherosclerosis and enabling quantification of anatomical and biological characteristics of subclinical atherosclerosis in a high risk population.

### Limitations

A major limitation of the study is the lack of blinding of study subjects, which is not possible due to the obvious nature of the intervention. This study has a small sample size, which may prove underpowered to achieve secondary objectives.

## Conclusion

In conclusion, the Baptist Employee Healthy Heart Study has been designed to investigate subclinical atherosclerosis using advanced noninvasive cardiovascular imaging in an asymptomatic high-risk population and personalized internet-based interventions to improve cardiovascular disease prevention in the early stages of atherosclerosis.

## Abbreviations

BEHHS, Baptist Employee Healthy Heart Study; BHSF, Baptist Health South Florida; CAC, coronary artery calcium; cIMT, carotid intima-media thickness; CVD, cardiovascular disease; MetS, metabolic syndrome; RHI, reactive hyperemia index
